# Phosphogenesis in the 2460 and 2728 million-year-old banded iron formations as evidence for biological cycling of phosphate in the early biosphere

**DOI:** 10.1002/ece3.443

**Published:** 2013-01-10

**Authors:** Yi-Liang Li, Si Sun, Lung S Chan

**Affiliations:** Department of Earth Sciences, The University of Hong KongHong Kong, China

**Keywords:** Banded iron formation, great oxidation event, iron oxide, phosphate, Precambrian, primary productivity

## Abstract

The banded iron formation deposited during the first 2 billion years of Earth's history holds the key to understanding the interplay between the geosphere and the early biosphere at large geological timescales. The earliest ore-scale phosphorite depositions formed almost at ∼2.0–2.2 billion years ago bear evidence for the earliest bloom of aerobic life. The cycling of nutrient phosphorus and how it constrained primary productivity in the anaerobic world of Archean–Palaeoproterozoic eons are still open questions. The controversy centers about whether the precipitation of ultrafine ferric oxyhydroxide due to the microbial Fe(II) oxidation in oceans earlier than 1.9 billion years substantially sequestrated phosphate, and whether this process significantly limited the primary productivity of the early biosphere. In this study, we report apatite radial flowers of a few micrometers in the 2728 million-year-old Abitibi banded iron formation and the 2460 million-year-old Kuruman banded iron formation and their similarities to those in the 535 million-year-old Lower Cambrian phosphorite. The lithology of the 535 Million-year-old phosphorite as a biosignature bears abundant biomarkers that reveal the possible similar biogeochemical cycling of phosphorus in the Later Archean and Palaeoproterozoic oceans. These apatite radial flowers represent the primary precipitation of phosphate derived from the phytoplankton blooms in the euphotic zones of Neoarchean and Palaoeproterozoic oceans. The unbiased distributions of the apatite radial flowers within sub-millimeter bands do not support the idea of an Archean Crisis of Phosphate. This is the first report of the microbial mediated mineralization of phosphorus before the Great Oxidation Event when the whole biosphere was still dominated by anaerobic microorganisms.

## Introduction

The Darwinian evolution of ecosystems at geological timescales is virtually coordinated by the evolving geological processes (Schwartzman et al. 1993; Nisbet and Sleep 2001; Riding 2002). For example, the amalgamation of 6 supercontinents from >2.65 billion years ago (Byr) to ∼350 million years ago (Myr) was coincident with the 6-step rises of atmospheric oxygen by enhanced releasing of nutrients to the marine ecosystems (e.g., Campbell and Allen 2008). Phosphorus is a vital nutrient element for the whole living world for making genetic materials, most of coenzymes and biochemical energy carriers (Westheimer 1987). The feedback mechanisms between geo-environment, ecosystem and climate that regulate the evolution of the whole Earth system can be traced by the sedimentary geology of phosphate through the deep time (Föllmi 1996; Föllmi et al. 2004). The geochemical cycle of phosphorus is a critical factor that governs the primary productivity of the aquatic ecosystems since the beginning of biological evolution (Geider et al. 2001; Bekker et al. 2010). For the first 2 billion years of Earth with life, banded iron formations (BIF) are the most important sedimentary rocks (Ohmoto et al. 2006; Bekker et al. 2010) holding key evidence for biological cycling of phosphorus and bioproductivity (Cloud 1973; Bjerrum and Canfield 2002; Kappler et al. 2005). Hematite and magnetite in BIFs are Fe^3+^- and [Fe^2+^,Fe^3+^]-bearing oxides representing mineralizations resulted from photoautotrophic and heterotrophic metabolisms (Konhauser et al. 2002, 2007a). As the major sedimentary rock of early Precambrian eons, BIFs have been reported to yield petrochemical, organic geochemical and isotopic evidence for microbial cycling of Fe, carbon, phosphorus, and nitrogen (e.g., Baur et al. 1985; Widdel et al. 1993; Bjerrum and Canfield 2002; Konhauser et al. 2002; Godfrey and Falkowski 2009). However, the mineral record of biological cycling of phosphorus has not yet been evidenced through the BIF-depositional history.

Phosphate is a common petrochemical component of BIF (e.g., Morris 1993; Bekker et al. 2010), but its mineralogy, such as crystal size, distribution and genetic link to the other minerals in the BIF assemblage, is poorly understood. One reason is that the comparison of phosphogenesis in BIF with the younger sedimentary rocks such as those after Mesoproterozoic is difficult because of the significantly evolved sedimentary environment and the biosphere (e.g., Papineau 2010). Among the various biomineralization mechanisms, phosphatization was not an important way of fossilization until the emergence of the early animal life (Cook and Shergold 1984; Valentine 2002; Luo 2005; Pyle et al. 2006). In the process of phosphatization, the microbial activities not only accelerate the phosphorization of soft tissues of the animal life (e.g., Wilby et al. 1996), it may also directly mediate the precipitation of phosphate (Sánchez-Navas and Martín-Algarra 2001; Schulz and Schulz 2005). However, phosphatization in a pure microbial environment, especially those in the anaerobic world before the bloom of aerobic respiration, is still poorly understood due to the rare fossil records or biomineralizations (e.g., Han and Runnegar 1992; Kolo et al. 2009). The onset of economic-scaled phosphorite deposition at about 1.9–2.2 Gyr (e.g., Banerjee 1971) after the Great Oxidation Event (GOE) at ∼2.45 Gyr indicated that the oxidizing of the atmosphere enhanced the flux of mineral nutrients from the terrestrial weathering and consequently increased aerobic primary productivity (Xiong and Bauer 2002; Bekker et al. 2004; Campbell and Allen 2008; Kirschvink and Kopp 2008; Papineau et al. 2009; Papineau 2010). However, the anaerobic world before GOE was probably very different with reduced atmosphere and ocean when the planetary ecosystem was dominated by anaerobes whose primary production was at least an order of magnitude less than that of today (e.g., Martin et al. 2003; Battistuzzi et al. 2004; Kharecha et al. 2005; Canfield et al. 2006). In the Neoarchean and Palaeoproterozoic oceans, the photosynthetic oxidation of dissolved Fe(II) was the result of the major biogeochemical process (Canfield et al. 2006; Konhauser et al. 2007a; Crowe et al. 2008; Bird et al. 2011). As the result, the planetary wide and secular deposition of iron oxides (e.g., Morris 1993) could be a major factor that constrained the primary productivity in the ocean. It has widely been observed that the oxidation of Fe(II) in the aquatic geochemical environments leads to precipitation of iron oxide fine particles that strongly absorb phosphorus and fix it to the sediments (e.g., Berner 1973; Krom and Berner 1980; Wheat et al. 1996; Poulton and Canfield 2006). Bjerrum and Canfield (2002) therefore proposed a phosphorus crisis hypothesis for the pre-1.9 Gyr oceans that the planetary wide precipitation of ferric iron oxides might have significantly sequestrated phosphorus from the ocean and limited the primary productivity to almost 25% of the modern marine biosphere. Experimental results by Konhauser et al. (2007b) argued that the phosphorus crisis could not happen because the dissolved silica can preferably occupy the absorbing sites on iron oxides that might have saved phosphorus for biological cycling.

In the early Precambrian ocean, the upwelling zone was the redox interface with concentrated biological activity and precipitation of minerals including biogenic phosphate (Morris 1993; Papineau 2010). Li et al. (2011) recently reported the overgrowth of 40–60 nm apatite and likely biogenic magnetite in the 2480-Myr BIF from Dales Gorge of Western Australia that suggests the existing of Fe(III)-respiring heterotrophs beneath the photoautrophs in the euphotic zone. However, there is still no evidence that supports a similar phosphogenesis at the beginning and the end of Proterozoic: one was the transition from the anaerobic to the aerobic word (e.g., Kirschvink and Kopp 2008; Papineau 2010) and the other was the emergence of animal life. In this study, we report the high similarity between the microbial phosphogenesis in the 535-Myr Lower Cambrian phosphorite and those in the 2460-Myr Kuruman and the 2728-Myr Abitibi BIFs. This observation comparably suggests the biogenecity of the observed apatite in BIFs and also indicates that the precipitation of iron oxides did not limit the biological cycling of phosphorus in the early Precambrian oceans.

## Samples

### Lower Cambrian phosphorite

The Lower Cambrian siliceous phosphorite is a ∼0.2–0.5 m layer overlaying the fossil rich Dengying Group dolomitite (635–551 Myr, e.g., McFadden et al. 2008; Condon et al. 2005); it is conformably overlain by the polymetallic Ni-Mo-PGE-Au layer of about the same thickness in the Niutitang Formation black shale with an Re-Os isochron age of 535 ± 11 Myr (Jiang et al. 2007). The Ni-Mo-PGE-Au layer is well known for its extremely high contents of Ni-sulfides (peak value 7.5 wt%) and Mo-sulfide (peak value 3.8 wt%) with controversial forming mechanism (Steiner et al. 2001; Jiang et al. 2007). The Niutitang Formation was overlain by strata with Chengjiang Fauna (Hou et al. 1991, 2004) of about ten million years younger (∼525 Myr). The phosphorite layer represents an accumulation of biological cycled phosphorus in the middle of Cambrian explosion.

### Kuruman iron formation

The Palaeoproterozoic Kuruman BIF of Transvaal Group in the Northern Cape Province, South Africa was dated by SHRIMP U-Pb zircon chronology to be 2460 Myr, (Pickard 2003). The studied sample is from the rhythmical chert and oxide-rich bands of Groenwater member (Pickard 2003), which represents a type of shallowing-upward deposition from the deepwater (Beukes 1984). The peak metamorphic temperatures for Kuruman Iron Formation should not exceed 110–170°C (Miyano and Beukes 1984). The Kuruman Iron Formation deposited at the peak-time of BIF (Klein 2005) that was coincident with the transition from the anaerobic atmosphere to the oxygenic atmosphere that fundamentally changed the pathway of life evolution on Earth (Bekker et al. 2004).

### Abitibi banded iron formation

The Abitibi BIF is embedded in the uppermost section of the Hunter Mine Group of bimodal volcanic complex in the Abitibi greenstone belt, with an age of 2728 Myr (Mueller and Mortensen 2002). The sample for this study is from the chert-jasper-magnetite facies preserved in a large, folded rip-up clast within volcanic breccias (Chown et al. 2000; Weiershäuser and Spooner 2005). Chown et al. (2000) suggested that the iron facies banded iron represents normal pelagic sediment deposited in periods of volcanic quiescence. The Abitibi BIF deposited at Neoarchean with probably the first emergence of oxygenic cyanobacteria (e.g., Brocks and Banfield 2009), which could be also the first transition of biological cycling of carbon and nutrients of the early biosphere (Nisbet and Nisbet 2008).

### Methods

Electron microscopic observations were conducted at Electron Microscope Center of the University of Hong Kong. To prevent contamination, the cm-sized specimens were first polished to get surface roughness near ∼200 nm and then the edges were bladed with a screwdriver to peel off thin flakes to produce new surfaces for immediate and direct observations using a Hitachi S4800 scanning electron microscope (SEM). Secondary electron (SE) modes at low voltage (3–5 kV) and high voltage (20 kV) were used for micrograph imaging and chemical composition analyses with equipped energy dispersive X-ray spectroscopy (EDS). A Philips Tecnai G2 20 S-TWIN scanning transmission electron microscope (STEM) equipped with selected area electron diffraction (SAED) and EDS was used for the high-resolution imaging of the mineral morphology and characterization of crystallographic structures. The organic matter in the Lower Cambrian phosphorite was extracted and analyzed for n-alkane, cyclo-alkane, and hopanes by gas chromatography – mass spectrometer (GC-MS) in the China University of Geosciences at Wuhan using the methods described by Xie et al. (2005). Room-temperature ^57^Fe Mössbauer spectroscopy of phosphorite was performed at the Department of Earth Sciences, the University of Hong Kong.

## Results

### Mineralogy, organic geochemistry, and apatite radial flowers of the 535-Myr phosphorite

**Figure 1:**
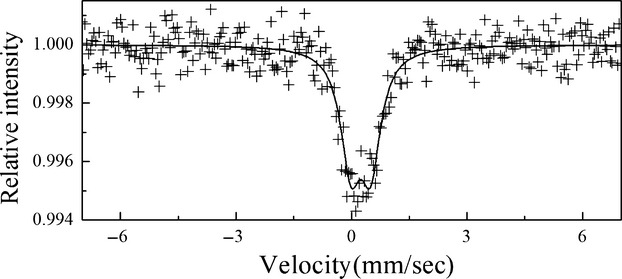
The ^57^Fe Mössbauer spectroscopy of the 535-Myr Lower Cambrian phosphorite indicating all ferric iron in the siliceous mineral structure.

**Figure 2:**
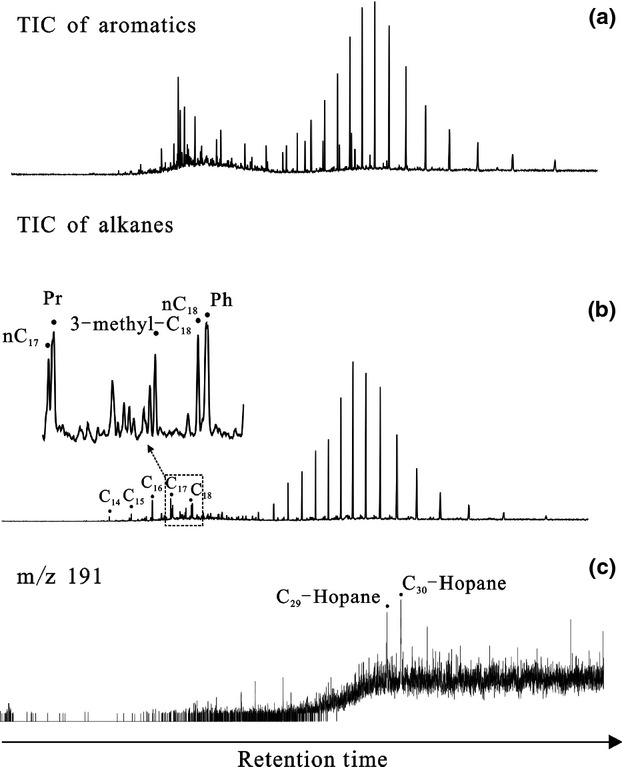
Profiles of (a) aromatic alkane, (b) n-alkane, and (c) hopanes detected in the 535-Myr phosphorite.

**Figure 3:**
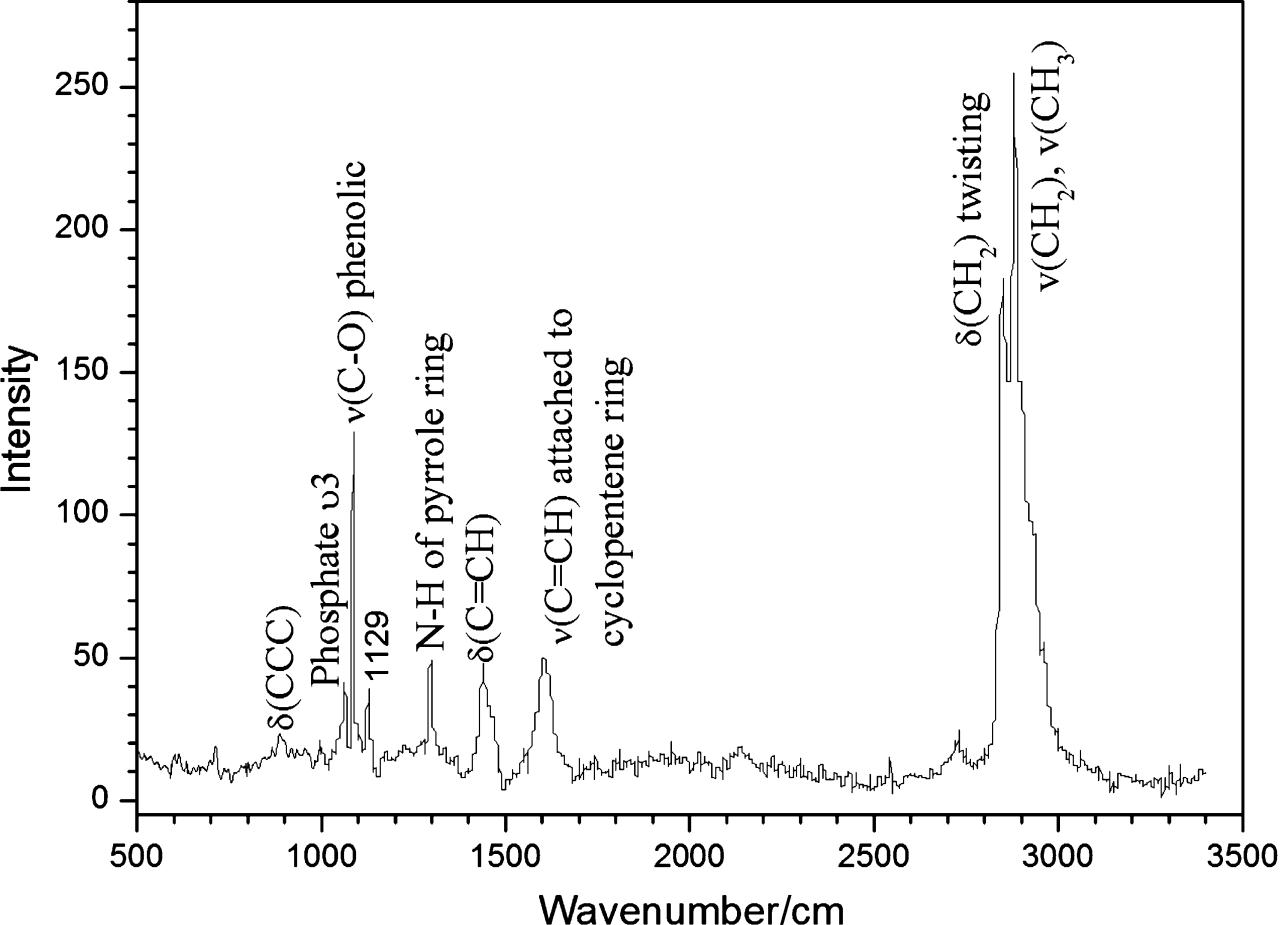
The Raman absorption bands of 888, 1090, 1440, and 1605 cm^−1^ synthetically showing the existence of scytonemin, the pigment only produced by oxygenic cyanobacteria to protect them from ultraviolet radiation.

The Mössbauer spectroscopic analysis showed that all measurable iron in phosphorite is paramagnetic ferric iron in the octahedral sites of phyllosilicate (IS = 0.24 mm/s and QS = 0.52 mm/s; Fig.1). The GC-MS analyzed organic extractions from phosphorite showed rich fossil molecules (Fig.2). The n-alkane profile showed carbon numbers nC12–nC38 without odd–even preference and a peak at nC16–nC18 (Fig.2a) implying major contribution of algal or microbial biomass. The ratio of pristane to phytane was 0.92 (Fig.2b) indicating a neutral to slightly reduced environment (Volkman et al. 1998). The low but detectable 3-methyl-heptadecane (Fig.2b) and C29- and C30-hopanes (Fig.2c) imply the existence of cyanobacteria (e.g., Summons et al. 1988). The Raman spectroscopic measurements showed the existence of δ(CCC), ν(C-O) phenolic, δ(C=CH), and ν(C=CH) attached to cyclopentene ring vibrations (Fig.3), which together indicates the existence of scytonemin, the pigment only produced by oxygenic cyanobacteria to filter ultraviolet radiations (Edwards et al. 1999).

**Figure 4:**
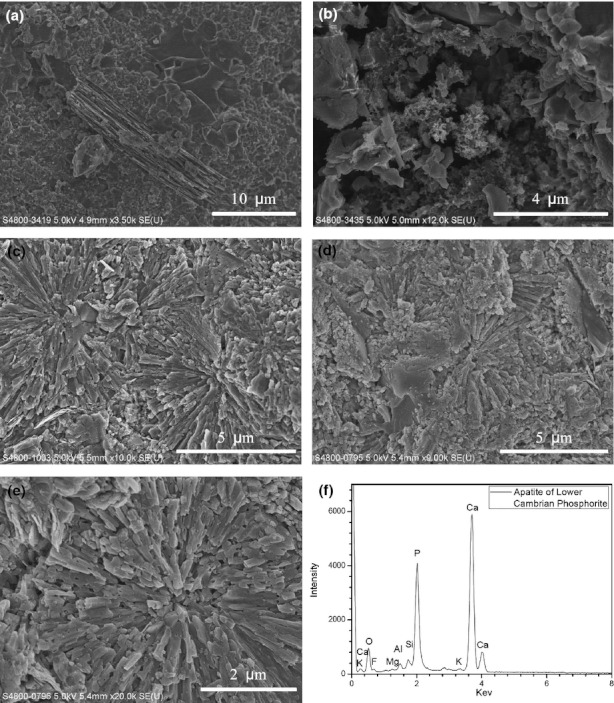
Phosphate aggregates in the 540-Myr Lower Cambrian Phosphorite. (a) Kaolinite and amorphous SiO_2_. (b) Organic matter mixed with opal-A type silica; (c-e) Apatite radial flowers. Particularly, the high-resolution image in Fig.4e showing radial structure of compacted apatite crystals and porosities in the amorphous phosphate composition. (f) EDS analysis showing a phosphate composition.

The phosphorite contains small fractions of crystallized and amorphous SiO_2_, kaolinite (Fig.4a), and a mixture made of organic carbon and amorphous silica (Fig.4b). Phosphate (apatite) appearing as radial flowers (ARF) is the most abundant mineral in phosphorite with their sizes ranging 3–7 μm. The rays of ARF are made of a core of crystallized apatite and the skin of amorphous phosphate that cements the rays together (Fig.4e). These ARFs account for >50% of the mineral compositions of phosphorite. The EDS analyses (Fig.4f) indicated that both the apatite crystals and the amorphous substances are fluorapatite in composition. However, structural sulfate and carbonate in apatite were also detected.

**Figure 5:**
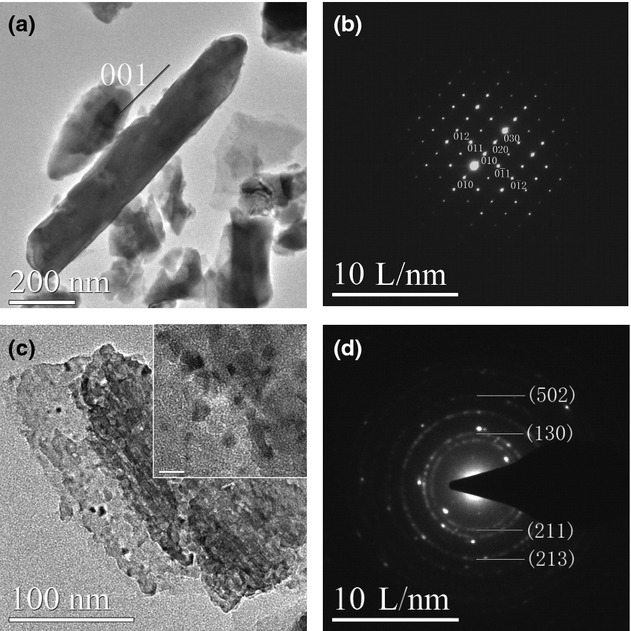
Characterization of the single crystal of apatite from Lower Cambrain phosphorite. (a) The long axis of the single crystal is (001) or *c* axis. (b) SAED pattern shows the structure of apatite. (c) The amorphous part between the crystallized apatite rays is also phosphate in composition with scattered crystallized domains. (d) SEAD pattern showing these crystallized domains as powder of apatite ultrafine particles.

The high abundance of phosphate particles in phosphorite allows characterization of their structures by STEM (Fig.5). The long-axis direction of the phosphate rod is determined to be {001} or *c* axis (Fig.5a), which is perpendicular to its Miller index (001) determined by SAED (Fig.5b). The amorphous phosphate skin wrapped the crystallized rod appears to be thin foil under STEM (Fig. 5c and inset), which shows sporadically distributed much finer spots with crystal fringe (<5 nm) at high resolution (inset of Fig.5c). When these crystallized domains served as randomly distributed phosphate crystallites, they actually produced diffraction pattern of Ca-apatite (Fig. 5d). Although only ARFs from the 535-Myr phosporite were determined for mineral structures, the same structure and crystallographic habits of ARFs from the Abitibi- and Kuruman-BIFs could be extrapolated based on their similar chemical composition and morphology.

### Apatite radial flowers in the Kuruman BIF

**Figure 6:**
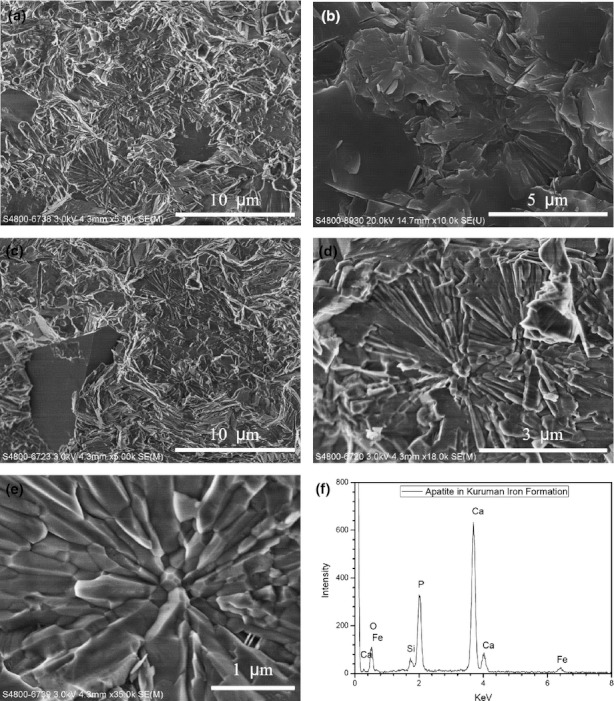
Phosphate aggregates in the 2460-Myr Kuruman iron formation. (a) ARFs in the matrix of jasperous material. (b) Two ARFs observed under high voltage (25 keV). (c) ARFs coexisting with magnetite crystal. (d) The amplification of ARFs in (c). (e) High-resolution image showing radial structure of compacted apatite crystals. (f) EDS analysis showing the phosphate composition.

In the 2460-Myr Kuruman BIF, ARFs were only observed in a few microbands. The sizes of ARFs are similar to those in the Lower Cambrian phosphorite (Fig. 6a–d). Hematite (dark part, Fig.6b) and euhedral magnetite crystals of a few tens of μm (Fig.6c), and ferro-dolomite crystals of a few 10 μm (not shown) were observed coexisting with ARFs. Figure 4e showed a particular ARF made of submicrometer crystal rods tightly compacted with each other, however, without amorphous cementing phosphate that ARFs of the Lower Cambrian phosphorite have. The composition of those ARFs is Ca_3_PO_4_ as analyzed by EDS (Fig. 6f).

### Apatite radial flowers in the Abitibi BIF

**Figure 7:**
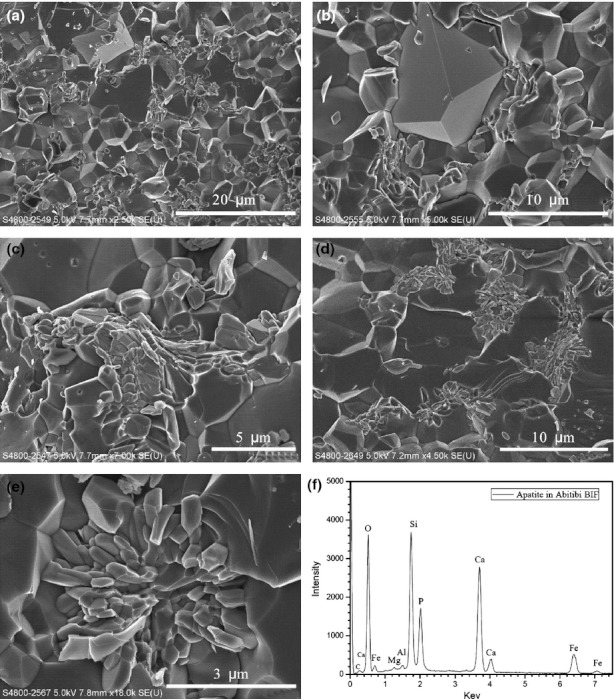
Phosphogenesis in the 2728-Myr banded iron formation from Abitibi, Canada. (a) The coexisting of ARFs, euhedral magnetite crystal in the matrix of chert polyhedrons. (b) A close observation of the coexisting of ARFs, magnetite and chert. (c) ARF with flattened appearance. (d) ARFs coexisting with chert and hematite breccias. (e) A particular ARF structure; (f) EDS analysis indicating a phosphate composition.

For the Abitibi BIF, both chert- and iron oxides-rich bands were found to have relatively abundant ARFs when compared with the Kuruman BIF samples. Due to the relatively high-grade metamorphic process the sample experienced, silica in both chert bands and the iron-oxide-rich bands appears as aggregates of well-shaped polyhedrons with their average sizes of 6.2 ± 2.3 μm, based on a statistics of 32 grains on one SEM micrograph of 21 × 16 μm^2^ (Fig. 7a). Petrologically, the magnetite-rich thin layers do not show visible changes after metamorphism, similar to that previous reported by Chown et al. (2000). Magnetite crystals in both silica- and iron-rich bands are similar in size and morphology of a few tens of μm (Fig.5b). Hematite breccias were found to coexist with either magnetite or silica with their sizes as large as >100 μm (Fig.7c). Similar to ARFs in the 535-Myr phosphorite and the 2460-Myr BIF, ARFs in the 2728-Myr BIF have almost the same sizes ranging 3.5–7.0 μm (Fig.7a–e). These ARFs can be observed scattered around silica polyhedrons (Fig.5a), magnetite crystals (Fig.7a–b), and hematite breccias (Fig.7c–d). Figures 7c–d show that the structure of ARFs was flattened rather than spherules due probably to compaction and greenschist grade metamorphic pressures (Chown et al. 2000). Similar to the Kuruman ARFs, those in the Abitibi BIF are also made of compacted rod-shaped apatite with clear boundaries to each other (Fig.7e) and also with a chemical composition of Ca_3_PO_4_ (Fig.7f).

## Discussion

These ARFs are different from the big, round phosphate grains of ∼20 μm with metamorphic genesis (Trendall & Blockley, #b^201^; Nutman and Friend 2006) observed in the ore bodies hosted by BIF, which experienced supergene metasomatism during Mesozoic-Tertiary (Dukino et al., #b^202^). The submicrometer crystal sizes and a few μm of ARFs coexisting with varied minerals in these BIF samples only experienced low-grade metamorphism that suggests a possible authigenic nature, as previously suggested by Ehrlich (1990).

The existence of amorphous calcium phosphate among the crystallized rays in ARFs of the 535-Myr phosphorite indicates the precipitation of the phosphatic gel-precursor under supersaturated condition at the early diagenetic stage (e.g., Sánchez-Navas and Martín-Algarra 2001). The high similarities of the structure and chemical composition of ARFs imply a similar diagenetic history experienced by the Kuruman- and Abitibi-ARFs. Comparatively, the porous textures on the rays of ARFs of the 535-Myr phosphorite (Fig.4e) resemble bacterial growth structures previously described (Martín-Algarra and Sánchez-Navas 2000). The abundances of ARFs in the Abitibi and Kuruman BIFs are too low to allow TEM-based structure characterization. The high similarities of genetic pattern of ARFs, morphology and chemical composition of apatite crystals from Precambrian BIFs to those in the Cambrian Phosphorite suggest that they have the same mineralogical structure (Meldrum and Cölfen 2008). ARFs of Abitibi and Kuruman all show well-crystallized habits, probably due to their long aging time and the greenschist grade metamorphisms. The jaspilitic matrix is well preserved in the Kuruman BIF, but is overprinted in the Abitibi BIF due to the slightly higher grade metamorphism (e.g., Weiershäuser and Spooner 2005). The 40–60 nm apatite crystal size in a matrix of the same composition (Li et al. 2011) overgrowth with likely biogenic magnetite in the 2.48-Gyr Dales Gorge BIF was probably presented during the even earlier stage of crystallization, which also survived the diagenetic process (Sánchez-Navas and Martín-Algarra 2001).

The similarity of phosphogenesis and composition of ARFs preserved across such a long geological time enable a better understanding of the early Precambrian phosphates because the lithology of phosphorites after Cambrian is commonly accepted as a biosignature (Xiao and Knoll 2000; Föllmi et al. 2004). Similar ARFs formed in the sulfidic ocean of Cambrian and the ferruginous oceans of 2728 Myr and 2460 Myr indicated that iron oxide precipitation in the BIF-depositional oceans did not influence the cycling of phosphorus (Fig.4a) due probably to the dominating of oxygenic cyanobacteria in primary productivity during this time (Knoll 2008). The depositions of phosphorite in the Lower Cambrian black shale indicated a short period of oxidized environment (McFadden et al. 2008), which can be corroborated by the 100% Fe(III) in the coexisting of kaolinite, hopane, and scytonemin that indicated existence of oxygenic photosynthetic community (Fig.2, Fig.3). However, the Pr/Ph ratio indicated a relatively reducing environment (Volkman et al. 1998). These apparently conflicting redox indicators suggest a possible redox interface environment, such as the upwelling zone. It is inferred that these BIF hosted ARFs of the early Precambrian eons also precipitated in relatively oxidized environments with the thriving of oxygenic photosynthesis. Besides, ARFs widely observed coexisting with different minerals in the early Precambrian BIFs also indicate an extensive biological mediation (Morris 1973) rather than a scenario with strong iron oxide removal of phosphorus (Bjerrum and Canfield 2002) because there is no biased distribution of ARFs in hematite-rich bands, as observed by SEM. These observations support the conclusion from the experimental observation that the dissolved silica inhibited the absorption of phosphate to the precipitated iron oxides (Konhauser et al. 2007b; Planavsky et al. 2010). The abundance of ARFs in the Abitibi and Kuruman BIFs is much lower than that in the Lower Cambrian phosphorite, which, however, indeed implies the low primary productions in the Neoarchean – Paleoproterozoic oceans (Kharecha et al. 2005; Canfield et al. 2006).

The precipitation of phosphate from the aquatic ecosystem was biologically controlled (Stal 2000; Goldhammer et al. 2010), and that in modern marine environments, such as in the upwelling zone, can be the result of secular accumulation of episodically released organic phosphorus into the sediment by abundant benthic bacterial activities (Piper and Codispoti 1975; Schulz and Schulz 2005; Goldhammer et al. 2010). The similar microbial-mediated phosphogenesis in probably the similar environments was discovered in the Neoproterozoic phosphorite (Bailey et al. 2007), which was consistent with an oxidized Ediacaran ocean (Fike et al. 2006). It is inferred that the precipitation of ARFs in BIFs was also related to the relative oxidative environments where oxygenic photosynthetic bacteria thrived (Morris 1993; Bjerrum and Canfield 2002; Papineau 2010). If the enrichment of phosphate was the result of localized phytoplankton bloom, the marine environment for precipitation should be within the ancient upwelling zone (Morris 1993; Papineau 2010). Actually the shale normalized positive ytterium anomalies of the Kuruman Iron Formation indicates an oxygenated shelf environment with upwelling marine bottom water (Bau and Dulski 1996). The similarity of ARFs in the 2728-Ma and the 2460-Myr BIFs to the 535-Myr phosphorite provides strong evidence for an Neoarchean-Palaeoproterozoic oxidation environment that could be extrapolated from the Cambrian biological and geological evidences (e.g., Young et al. 1998; Olson 2006; Buick 2008).

## Conclusions

The high similarities of apatite radial flowers in banded iron formations of Neoarchean to Palaeoproterozoic eras to those in the Cambrian phosphorite with rich evidences of microbial activities suggest similar ecophysiologial environments. The coexisting of phosphate with hematite, magnetite, and jasperous matrix in banded iron formation shows that the secular and planetary wide precipitation of ferric iron oxides that resulted from microbial photosynthesis did not cause the phosphate crisis in the early Precambrian marine ecosystems. The dominance of anaerobic metabolism was probably the major reason for the low primary productivity of the Palaeoproterozoic and Neoarchean biospheres.

## References

[b1] Bailey JV, Joys SB, Kalanetra KM, Flood BE, Corsetti FA (2007). Evidence of giant sulfur bacteria in Neoproterozoic phosphorites. Nature.

[b2] Banerjee DM (1971). Precambrian stromatolitic phosphorites of Udaipur, Rajasthan, India. Geol. Soc. Am. Bull.

[b3] Battistuzzi FU, Feijao A, Hedges SB (2004). A genomic timescale of prokaryote evolution: insights into the origin of methanogenesis, phototrophy, and the colonization of land. BMC Evol. Biol.

[b4] Bau M, Dulski P (1996). Distribution of yttrium and rare-earth elements in the Penge and Kuruman iron-formations, Transvaal Supergroup, South Africa. Precambr. Res.

[b5] Baur ME, Hayes JM, Studley SA, Walter MR (1985). Millimeter-scale variations of stable isotope abundances in carbonates from banded iron-formations in the Hamersley Group of Western Australia. Econ. Geol.

[b6] Bekker A, Holland HD, Wang PL, Rumble D, Stein HJ, Hannah JL (2004). Dating the rise of atmospheric oxygen. Nature.

[b7] Bekker A, Slack JF, Planavsky N, Krapež B, Hofmann A, Konhauser KO (2010). Iron formation: the sedimentary product of a complex interplay among mantle, tectonic, oceanic, and biospheric processes. Econ. Geol.

[b8] Berner RA (1973). Phosphate removal from sea water by adsorption on volcanogenic ferric oxides. Earth Planet. Sci. Lett.

[b9] Beukes NJ (1984). Sedimentology of the Kuruman and Griquatown iron formations, Transvaal Supergroup, Griqualand West, South Africa. Precambr. Res.

[b10] Bird LJ, Bonnefoy V, Newman DK (2011). Bioenergetic challenges of microbial iron metabolisms. Trends Microbiol.

[b11] Bjerrum CJ, Canfield DE (2002). Ocean productivity before about 1.9 Gyr ago limited by phosphorus adsorption onto iron oxides. Nature.

[b12] Brocks JJ, Banfield J (2009). Unraveling ancient microbial history with community proteogenomics and lipid geochemistry. Nat. Rev. Microbiol.

[b13] Buick R (2008). When did oxygenic photosynthesis evolve?. Phil. Trans. R. Soc. B.

[b14] Campbell IH, Allen CM (2008). Formation of supercontinents linked to increases in atmospheric oxygen. Nat. Geosci.

[b15] Canfield DE, Rosing MT, Bjerrum C (2006). Early anaerobic metabolism. Phil. Trans. R. Soc. B.

[b16] Chown EH, N'hah E, Mueller WU (2000). The relation between iron-formation and low temperature hydrothermal alternation in an Archean volcanic environment. Precambr. Res.

[b17] Cloud P (1973). Paleoecological significance of the banded iron-formation. Econ. Geol.

[b18] Condon D, Zhu M, Bowring S, Wang W, Yang A, Jin Y (2005). U-Pb ages from the Neoproterozoic Doushantuo Formation, China. Science.

[b19] Cook PJ, Shergold JH (1984). Phosphorus, phosphorites and skeletal evolution at the Precambrian-Cambrian boundary. Nature.

[b20] Crowe SA, Jones CA, Katsev S, Magen C, O'Neill AH, Sturm A (2008). Photoferrotrophs thrive in an Archean Ocean analogue. Proc. Natl Acad. Sci. USA.

[b202] Dukino RR, England BF, Kneeshaw M (2000). Phosphorus distribution in BIF-derived iron ores of Hamersley Province, Western Australia. Appl. Earth Sci.

[b21] Edwards HGM, Garcia-Pichel F, Newton EM, Wynn-Williams DD (1999). Vibrational Raman spectroscopic study of scytonemin, the UV-protective cyanobacterial pigment. Spectrochimica Acta Part A.

[b22] Ehrlich HL (1990). Geomicrobiology.

[b23] Fike DA, Grotzinger JP, Pratt LM, Summons RE (2006). Oxidation of the Ediacaran ocean. Nature.

[b24] Föllmi KB (1996). The phosphorus cycle, phosphogenesis and marine phosphate-rich deposits. Earth-Sci. Rev.

[b25] Föllmi KB, Tamburini F, Hosein R, van de Schootbrugge B, Arn K, Rambeau C, Schneider SH, Miller JR, Crist E, Boston PJ (2004). Phosphorus, a servant faithful to Gaia? Biosphere remediation rather than regulation. Scientists Debate Gaia: the Next Century.

[b26] Geider RJ, Delucia EH, Palkowski PG, Finzi AC, Grime JP, Grace J (2001). Primary productivity of planet earth: biological determinants and physical constraints in terrestrial and aquatic habitats. Glob. Change Biol.

[b27] Godfrey LV, Falkowski PG (2009). The cycling and redox state of nitrogen in the Archaean ocean. Nat. Geosci.

[b28] Goldhammer T, Brüchert V, Ferdelman TG, Zabel M (2010). Microbial sequestration of phosphorus in anoxic upwelling sediments. Nat. Geosci.

[b29] Han TM, Runnegar B (1992). Megascopic eukaryotic algae from the 2.1-billion-year-old Negaunee iron-formation. Michigan Sci.

[b30] Hou X, Ramsköld L, Bergström J (1991). Composition and preservation of the Chengjiang fauna a Lower Cambrian soft-bodied biota. Zoolog. Scr.

[b31] Hou XG, Aldridge RJ, Bergström J, Siveter DJ, Feng XH (2004). The Cambrian fossils of Chengjiang, China: the flowering of early animal life.

[b32] Jiang S-Y, Yang J-H, Chen Y-Q, Feng H-Z, Zhao K-D, Ni P (2007). Extreme enrichment of polymetallic Ni-Mo-PGE-Au in Lower Cambrian black shales of South China: An Os isotope and PGE geochemical investigation. Palaeogeogr., Palaeoclimatol., Palaeoecol.

[b33] Kappler A, Pasquero C, Konhauser KO, Newman DK (2005). Deposition of banded iron formations by anoxygenic phototrophic Fe(II)-oxidizing bacteria. Geology.

[b34] Kharecha P, Kasting J, Siefert J (2005). A coupled atmosphere-ecosystem model of the early Archean Earth. Geobiology.

[b35] Kirschvink JL, Kopp RE (2008). Palaeoproterozoic ice houses and the evolution of oxygen-mediating enzymes: the case for a late origin of photosystem II. Phil. Trans. R. Soc. B.

[b36] Klein C (2005). Some Precambrian banded iron-formations (BIFs) from around the world: their age, geological setting, mineralogy, metamorphism, geochemistry, and origin. Am. Mineral.

[b37] Knoll AH, Herrero A, Flores E (2008). Cyanobacteria and Earth history. The Cyanobacteria; Molecular Biology, Genomics and Evolution.

[b38] Kolo K, Konhauser K, Krumbein WE, Hubin Y, van Ingelgem A, Claeys P (2009). Microbial dissolution of hematite and associated cellular fossilization by reduced iron phases: a study of ancient microbe-mineral surface interactions. Astrobiology.

[b39] Konhauser KO, Hamade T, Raiswell R, Morris RC, Ferris FG, Southam G (2002). Could bacteria have formed the Precambrian banded iron formations?. Geology.

[b40] Konhauser KO, Amskold L, Lalonde SV, Posth NR, Kappler A, Anbar A (2007a). Decoupling photochemical Fe(II) oxidation from shallow-water BIF deposition. Earth Planet. Sci. Lett.

[b41] Konhauser KO, Lalonde SV, Amskold L, Holland HD (2007b). Was there really an Archean phosphate crisis?. Science.

[b42] Krom MD, Berner RA (1980). Adsorption of phosphate in anoxic marine sediments. Limnol. Oceanogr.

[b43] Li YL, Konhuaser KO, Cole DR, Phelps TJ (2011). Mineral ecophysiological evidence for biogeochemical cycles in an early Paleoproterozoic banded iron formation. Geology.

[b44] Luo Z-X (2005). Doushantuo fossils: life on the eve of animal radiation. J. Paleontol.

[b45] Martin W, Rotte C, Hoffmeister M, Theissen U, Gelius-Dietrich G, Ahr S (2003). Early cell evolution, eukaryotes, anoxia, sulfide, oxygen, fungi first (?), and a tree of genomes revisited. IUBMB Life.

[b46] Martín-Algarra A, Sánchez-Navas A, Glenn CR, Lucas J, Prévôt-Lucas L (2000). Bacterially mediated authigenesis in Mesozoic stromatolites from condensed pelagic sediments (Betic Cordillera, southern Spain). Marine Authigenesis: from global to Microbial.

[b47] McFadden KA, Huang J, Chu X, Jiang G, Kaufman AJ, Zhou C (2008). Pulsed oxidation and biological evolution in the Ediacaran Doushantuo Formation. Proc. Natl Acad. Sci. USA.

[b48] Meldrum FC, Cölfen H (2008). Controlling mineral morphologies and structures in biological and synthetic systems. Chemical Review.

[b49] Miyano T, Beukes NJ (1984). Phase relations of stilpnomelane, ferriannite and riebeckite in very low-grade iron-formations. Trans. Geol. Soc. South Africa.

[b50] Morris RC (1973).

[b51] Morris RC (1993). Genetic modeling for banded iron-formation of the Hamersley Group, Pilbara Craton, Western Australia. Precambr. Res.

[b52] Mueller WU, Mortensen JK (2002). Age constrains and characteristics of subaqueous volcanic construction, the Archean Hunter Mine Group, Abitibi Greenstone belt. Precambr. Res.

[b53] Nisbet EG, Nisbet RER (2008). Methane, oxygen, photosynthesis, rubisco and the regulation of the air through time. Philos. Trans. R. Soc. B.

[b54] Nisbet EG, Sleep NH (2001). The habitat and nature of early life. Nature.

[b55] Nutman AP, Friend CRL (2006). Petrography and geochemistry of apatites in banded iron formation, Akilia, W. Greenland: consequence for oldest life evidence. Precambr. Res.

[b56] Ohmoto H, Watanabe Y, Yamaguchi KE, Naraoka H, Haruna M, Kakegawa T, Kesler SE, Ohmoto H (2006). Chemical and biological evolution of early Earth: constrains from banded iron formations. Evolution of early Earth's atmosphere, hydrosphere – constrains from ore deposits.

[b57] Olson JM (2006). Photosynthesis in the Archean Era. Photosynth. Res.

[b58] Papineau D (2010). Global biogeochemical changes at both ends of the Proterozoic: insights from phosphorites. Astrobiology.

[b59] Papineau D, Purohit R, Goldberg T, Pi D, Shields GA, Bhu H (2009). High primary productivity and nitrogen cycling after the Palaeoproterozoic phosphogenic event in the Aravalli Supergroup, India. Precambr. Res.

[b60] Pickard AL (2003). SHRIMP U-Pb zircon ages for the Palaeoproterozoic Kuruman Iron Formation, Northern Cape Province, South Africa: evidence for simultaneous BIF deposition on Kaapvaal and Pilbara Cratons. Precambr. Res.

[b61] Piper DZ, Codispoti AL (1975). Marine phosphorite deposits and the nitrogen cycle. Science.

[b62] Planavsky NJ, Rouxel OJ, Bekker A, Lalonde SV, Konhauser KO, Reinhard CT (2010). The evolution of the marine phosphate reservoir. Nature.

[b63] Poulton SW, Canfield DE (2006). Co-diagenesis of iron and phosphorus in hydrothermal sediments from the southern East Pacific Rise: implications for the evaluation of paleoseawater phosphate concentrations. Geochim. Cosmochim. Acta.

[b64] Pyle LJ, Narbonne GM, Nowlan GS, Xiao S, James NP (2006). Early Cambrian metazoan eggs, embryos, and phosphatic microfossils from northwestern Canada. J. Paleontol.

[b65] Riding R (2002). Microbial carbonates: the geological record of calcified bacterial-algal mats and biofilms. Sedimentology.

[b66] Sánchez-Navas A, Martín-Algarra A (2001). Genesis of apatite in phosphate stromatolites. Eur. J. Mineral.

[b67] Schulz HN, Schulz HD (2005). Large sulfur bacteria and the formation of phosphorite. Science.

[b68] Schwartzman D, McMenamin M, Volk T (1993). Did surface temperatures constrain microbial evolution?. Bioscience.

[b69] Stal LJ, Whitton BA, Potts M (2000). Cyanobacterial mats and stromatolites. The Ecology of Cyanobacteria.

[b70] Steiner M, Wallis E, Erdtmann B-D, Zhao Y, Yang R (2001). Submarine-hydrothermal exhalative ore layers in black shales from South China and associated fossils – insights into a Lower Cambrian facies and bio-evolution. Palaeogeogr., Palaeoclimatol., Palaeoecol.

[b71] Summons RE, Powell TG, Boreham CJ (1988). Petroleum geology and geochemistry of the middle Proterozoic McArthur Basin, Northern Australia: III. Composition of extractable hydrocarbons. Geochim. Cosmochim. Acta.

[b201] Trendall AF, Blockley JG (1970). The iron formation of the Precambrian Hamersley Group, Western Australia, with special reference to crocidolite. Geol. Sur. Western Australia Bull.

[b72] Valentine JW (2002). Prelude to the Cambrian Explosion. Annu. Rev. Earth Planet. Sci.

[b73] Volkman JK, Marrett SM, Blackburn SI, Mansour MP, Sikes EL, Gelin F (1998). Microalgal biomarkers: a review of recent research developments. Org. Geochem.

[b74] Weiershäuser L, Spooner ETC (2005). Seafloor hydrothermal fluids, Ben Nevis area, Abitibi Greenstone Belt: implications for Archean (∼2.7 Ga) seawater properties. Precambr. Res.

[b75] Westheimer FH (1987). Why nature choose phosphates. Science.

[b76] Wheat CG, Feely RA, Motti MJ (1996). Phosphate removal by oceanic hydrothermal processes: an update of the phosphorus budget in the oceans. Geochim. Cosmochim. Acta.

[b77] Widdel F, Schnell S, Heising S, Ehrenreich A, Assmus B, Schink B (1993). Ferrous iron oxidation by anoxygenic phototrophic bacteria. Nature.

[b78] Wilby PR, Briggs DEG, Bernier P, Gaillard C (1996). Role of microbial mats in the fossilliation of soft tissues. Geology.

[b79] Xiao S, Knoll A (2000). Phosphatization animal embryos from the Neoproterozoic Doushantuo Formation at Weng'an, Guizhou, South China. J. Paleontol.

[b80] Xie S, Pancost RD, Yin H, Wang H, Evershed RP (2005). Two episodes of microbial change coupled with Permo/Triassic faunal mass extinction. Nature.

[b81] Xiong J, Bauer CE (2002). Complex evolution of photosynthesis. Annu. Rev. Plant Biol.

[b82] Young GM, Gold V, von Brunn DJC, Minter WEL (1998). Earth's oldest reported glaciations: physical and chemical evidence from the Archean Mozaan Group (∼2.9 Ga) of South Africa. J. Geol.

